# Gallbladder-preserving polypectomy for gallbladder polyp by embryonic-natural orifice transumbilical endoscopic surgery with a gastric endoscopy

**DOI:** 10.1186/s12876-022-02269-y

**Published:** 2022-05-03

**Authors:** Xiao-Jian He, Zhi-Ping Chen, Xiang-Peng Zeng, Chuan-Shen Jiang, Gang Liu, Dong-Liang Li, Da-Zhou Li, Wen Wang

**Affiliations:** 1 Fuzhou General Clinical Medical College, Fujian Medical University, 156 North Road of West No.2 Ring, Fuzhou, 350025 China; 2 Department of Digestive Diseases, 900TH Hospital of Joint Logistics Support Force, Fuzhou, China; 3 Department of Digestive Diseases, Oriental Hospital Affiliated to Xiamen University, Fuzhou, China; 4 Department of Hepatobiliary Disease, 900TH Hospital of Joint Logistics Support Force, Fuzhou, China

**Keywords:** Gallbladder polyp, Gallbladder-preserving polypectomy, Embryonic-natural orifice transumbilical endoscopic surgery

## Abstract

**Background and aims:**

Cholecystectomy is performed for most gallbladder polyps (GPs). However, cholecystectomy results concerning complications in some patients. For benign GPs, adoption of gallbladder-preserving surgery is worth to recommend. We describe our experiences performing gallbladder-preserving polypectomy for GPs by embryonic-natural orifice transumbilical endoscopic surgery (E-NOTES) with a gastric endoscopy.

**Methods:**

This is a retrospective study of patients with GPs who underwent gallbladder-preserving polypectomy by E-NOTES with a gastric endoscopy from April 2018 to September 2019 in our hospital. The operative time, intraoperative hemorrhage, intraoperative and postoperative complications, gallbladder emptying function were obtained and analyzed.

**Results:**

The procedure was performed successfully in all 12 patients with 5 cases of single polyp and 7 cases of multiple polyps. The range of GPs size was 2 mm to 15 mm. The mean operation time was (95.33 ± 23.08) minutes (55–135 min). There were no adverse events including heavy bleeding, mortality and conversion to open surgery during operation. All patients were discharged in 4–5 days after surgery without postoperative complications such as delayed bleeding, fever, peritonitis, intra-abdominal abscess and abdominal wall incisional hernia. All patients were followed up at 1, 3, 6, and 12 months postoperation who had almost no visible incision on the umbilical region, no recurrent GPs. The gallbladder emptying function decreased one month after surgery, and gradually improved 3, 6 and 12 months after surgery.

**Conclusion:**

E-NOTES gallbladder-preserving polypectomy is a safe and effective option for patients with GPs and is close to scar-free surgery which can be performed in routine clinical practice.

## Introduction

Gallbladder polyps (GPs) are one of the most universal biliary diseases which are described as the gallbladder mucosa lesion protrudes into the gallbladder cavity. The estimated prevalence of GPs in adults ranges from 0.3 to 12.3% in varying parts of the world [1] and were identified in 6.9% in the Chinese population [2]. GPs can be divided into non-neoplastic polyps (including cholesterol polyps, inflammatory polyps, gallbladder adenomyomatosis) and neoplastic polyps (benign ones including adenoma, leiomyoma, lipoma, etc. malignant ones including adenocarcinoma) according to the pathological findings [3]. At present, the recognized risk factors for malignant GPs are: single polyp diameter more than 10 mm, combined with gallstone, fast-growing polyps, broad basal polyp, porcelain gallbladder, and patients older than 50 years, etc. [4].

GPs are often asymptomatic and diagnosed by abdominal ultrasound. However, it is still difficult to judge the benign and malignant polyps by abdominal ultrasound [5, 6]. Therefore, it is difficult to decide whether cholecystectomy is necessary for GPs. In fact, less than 5% of patients undergoing cholecystectomy for GPs have malignant lesions [7]. Pathological examination is still the gold standard for diagnosing the nature of GPs. At present, cholecystectomy is the main method for the treatment of GPs to obtain pathological diagnosis. However, surgical complications include bile duct injury, vascular injury, chronic diarrhea, etc., and the loss of postoperative gallbladder physiological function seriously affects the quality of life of patients [8, 9]. These shortcomings raise many specialists’ concerns about conservation of the gallbladder.

With the rise of minimally invasive endoscopic technology, the concept of embryonic-natural orifice transumbilical endoscopic surgery (E-NOTES) has been put forward and continuously developed [10]. Its route is through the umbilicus, the natural orifice of the embryo. Hence the incisions are hidden in the umbilicus, there is a "nearly scar-free" post-surgical bellybutton. In this study, we reported our initial experiences of E-NOTES for the treatment of GPs with gallbladder-preserving polypectomy.

## Materials and methods

### Subjects

12 patients with GPs at the 900th Hospital of PLA between April 2018 and September 2019 were enrolled. Inclusion criteria included patients with (a) aged 18–70 years; (b) abdominal color ultrasound suggested GPs, and the maximum size of GPs > 5 mm; (c) gallbladder wall thickness less than 4 mm; (d) accepting E-NOTES gallbladder-preserving polypectomy. Exclusion criteria included patients with (a) complicated with gallstone, acute or chronic cholecystitis, (b) no functional or malfunctioning gallbladder, (c) porcelain gallbladder, (d) poor diabetes control, (e) suffering from malignant tumors, severe heart, liver, or renal dysfunction and history of gastrointestinal surgery, (f) pregnancy and lactation, (g) psychiatric disorders.

### Surgical equipment

The instruments used in this study included gastric endoscopy (GIF-H260, Q260; Olympus Optical Co., Ltd., Tokyo, Japan), transparent cap (Olympus Medical Systems), insulation-tipped knife (KD-650L; Olympus Medical Systems), Hook knife (KD-620QR; Olympus Medical Systems), polypectomy snares (STIFFBx/10; Boston Scientific Corporation), hot biopsy forceps (KD-410LR; Olympus Medical Systems), hemostatic clamp (Boston Scientific Corporation), VIO 300D device (ERBE Elektromedizin GmbH, Germany), homemade Trocar. The gastroscope was cleaned and disinfected by the disinfectant center and then sterilized with ethylene oxide.

### Operative technique

**Fig. 1:**
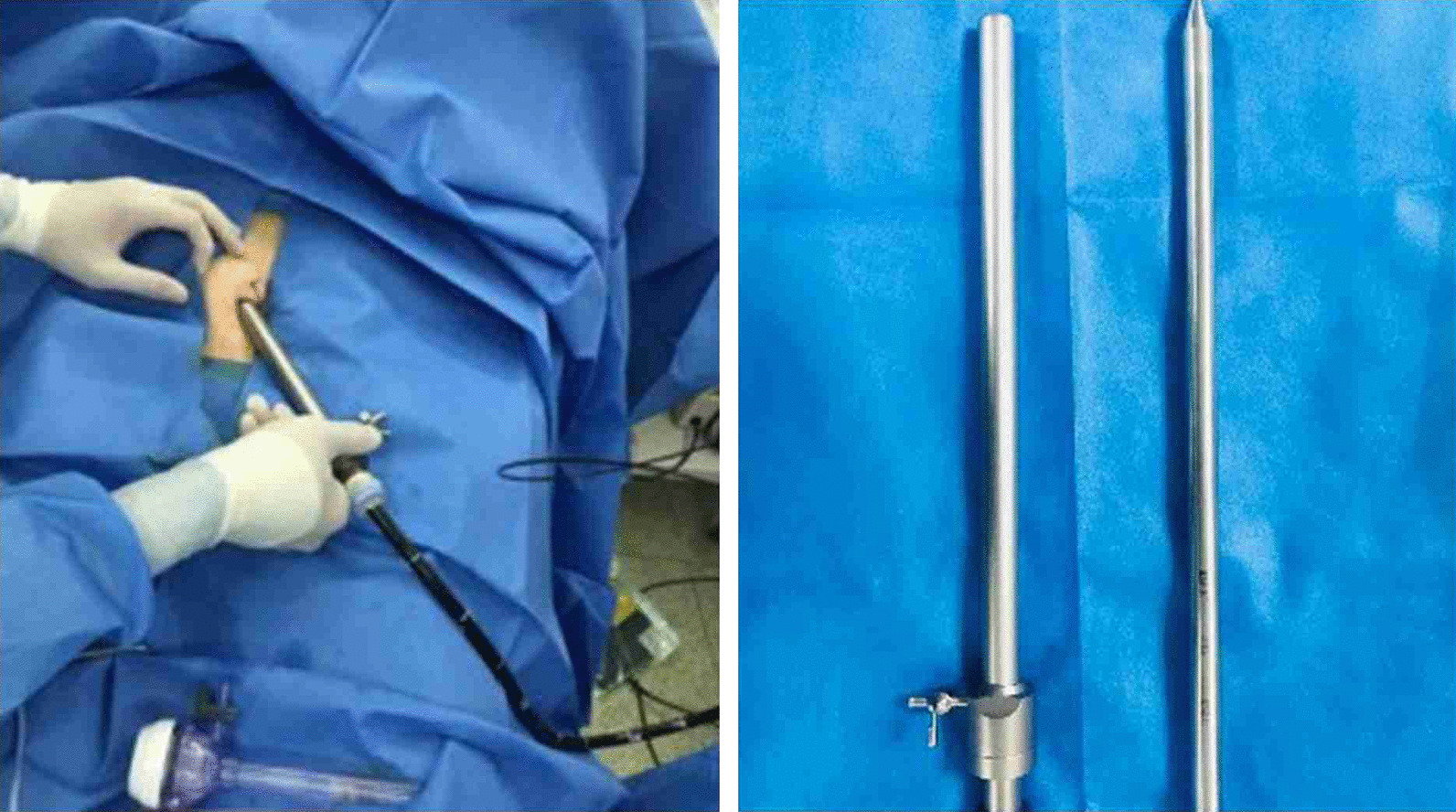
The gastric endoscopy was introduced into the abdominal cavity through a 10-mm homemade trocar

**Fig. 2:**
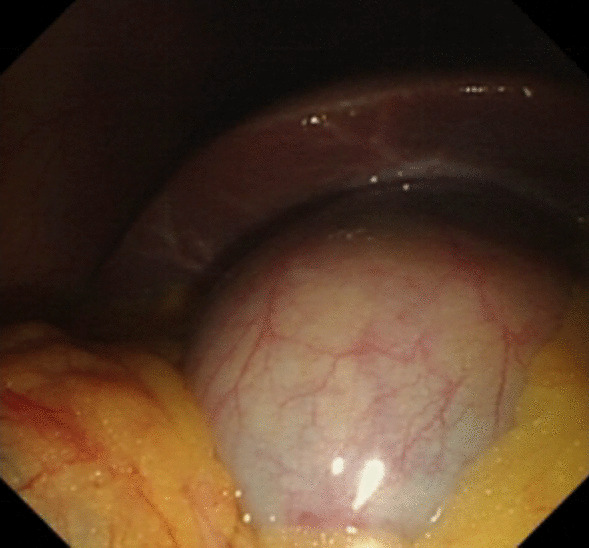
The appearance of the gallbladder under direct vision of the gastric endoscopy

**Fig. 3:**
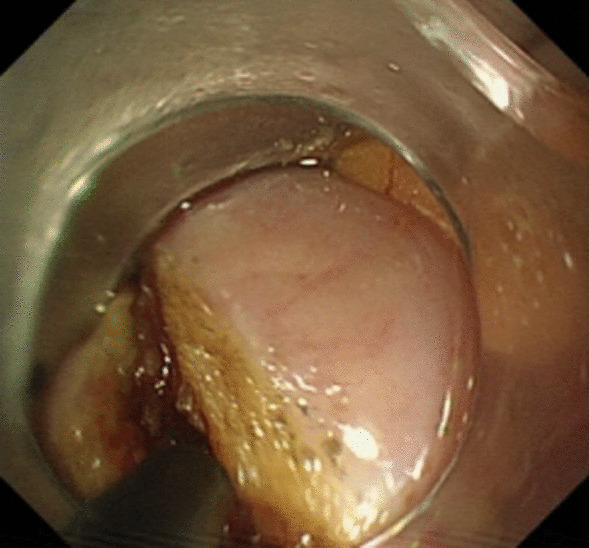
The gallbladder fundus was cut by IT or hook knife

**Fig. 4:**
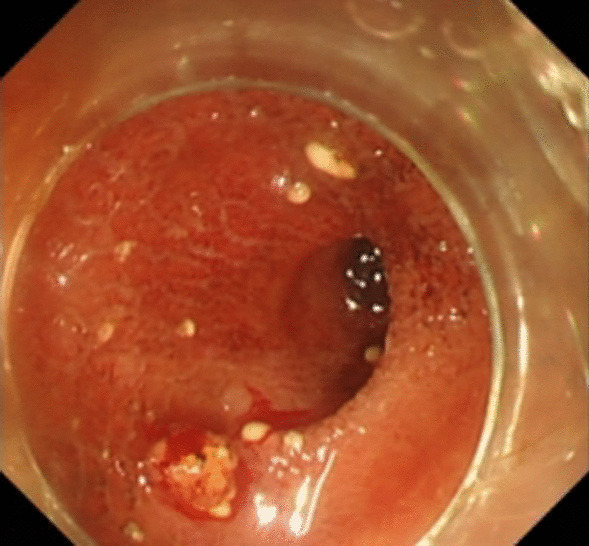
An endoscopic image of the gallbladder cavity and the different size and morphology of GPs

**Fig. 5:**
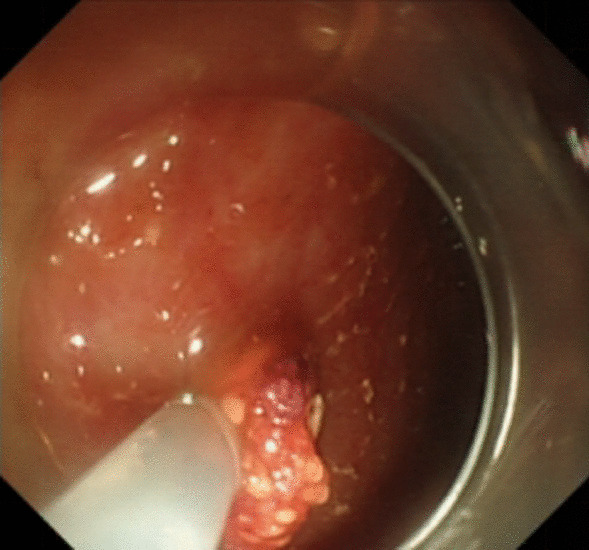
A snares was selected to completely remove the GPs (diameter > 5 mm)

**Fig. 6:**
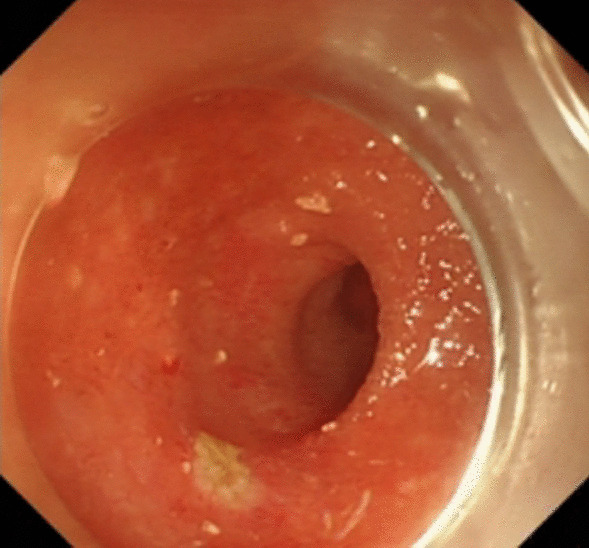
Argon plasma coagulation (APC) for the treatment of GPs

**Fig. 7:**
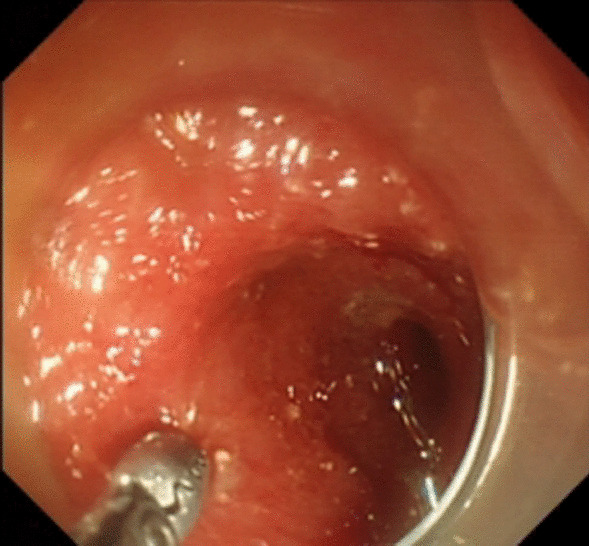
Biopsy forceps were used to bite the GPs (diameter ≤ 5 mm)

**Fig. 8:**
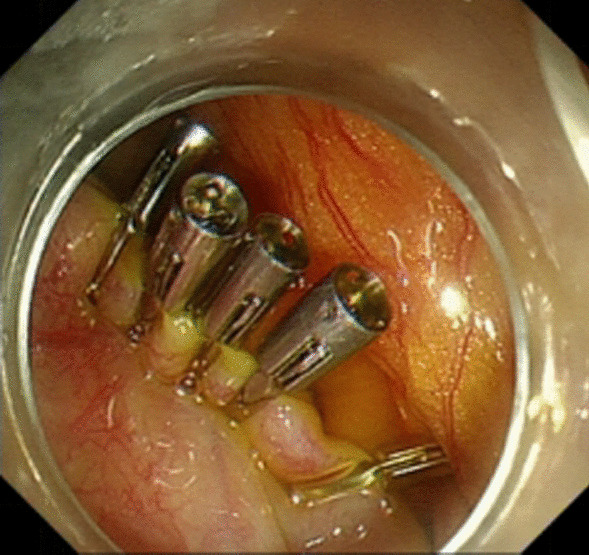
The gallbladder wall incision was closed with hemostatic clamps

**Fig. 9:**
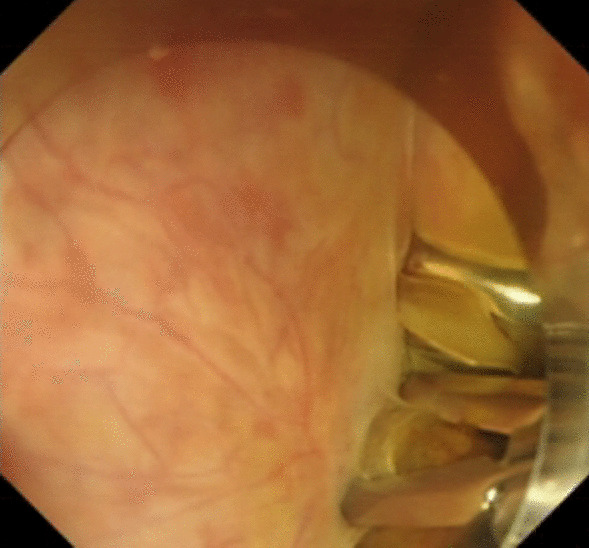
The abdominal cavity was rinsed with normal saline

**Fig. 10:**
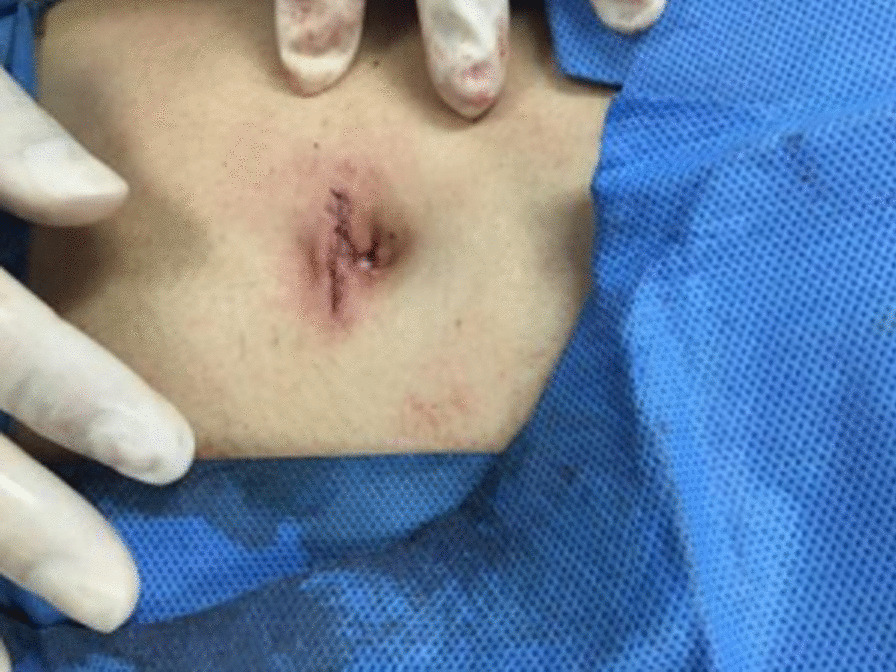
The umbilical incision was cosmetically closed

Under general anesthesia, the patient was placed supine. After the operating area was disinfected and covered with towel, a 10-mm trocar was inserted through the umbilicus (Fig. 1), and then the gastric endoscopy with a transparent cap was introduced to observe the abdominal cavity adhesions and the appearance of the gallbladder (Fig. 2). The gallbladder fundus was cut by IT or Hook knife (Fig. 3), and bile spilled out and was withdrawn. After entering the gallbladder cavity, treatment instruments were selected according to the size and morphology of GPs (Fig. 4). For diameter polyps larger than 5 mm, a snares or argon plasma coagulation (APC) was selected to completely remove the GPs (Figs. 5, 6,); for small polyps less than or equal to 5 mm, biopsy forceps were used to bite them (Fig. 7). The gallbladder cavity was flushed with normal saline to explore the gallbladder and eliminate the residual polyps. The gallbladder wall incision was closed with hemostatic clamps (Fig. 8). The abdominal cavity was rinsed with normal saline (Fig. 9). Finally, the umbilical incision was cosmetically closed (Fig. 10).

### Measurement of the gallbladder-emptying function

The gallbladder-emptying function was measured by abdominal ultrasound before surgery and 1, 3, 6 and 12 months after surgery which was performed on an empty stomach and 45 ~ 60 min after the fat meal (two fried eggs) to record the length, width and height of the gallbladder. The calculation formula of gallbladder volume is as follows: gallbladder volume (cm^3^) = π/6 × length × width × height (cm) [11, 12]. The gallbladder-emptying function was expressed by the gallbladder emptying index: (fasting volume—postprandial volume)/ fasting volume × 100%. The gallbladder-emptying function was good, acceptable, poor respectively when the gallbladder emptying index were more than 50%, 33.3% to 50%, less than 33.3% [13].

### Postoperative management and follow-up

The postoperative complications and vital signs were closely observed. Fluidized diet was administered 24 h after operation. Follow-up at 1, 3, 6, and 12 months after the operation included evaluation of clinical symptoms, umbilicus incision, recurrent GPs, and gallbladder-emptying function.

### Statistical analysis

Measurement data were expressed as mean ± standard deviation. Matched-pair analysis was used to compare the data of gallbladder volume and gallbladder-emptying function before and after surgery. SPSS 26.0 statistical software (SPSS, Inc., Chicago, IL) was used to analyze all the study data and draw the linear graph.

## Results

### Operative procedures

**Fig. 11:**
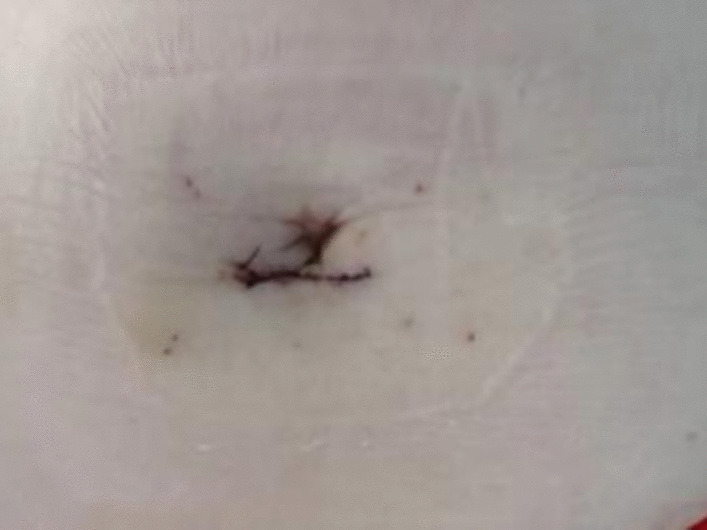
The umbilical incision healed well after operation

In this study, there were 12 patients, including 8 males and 4 females, with a median age of (47.33 ± 8.12) years (36–62 years). All the operations were successful, and the mean procedure time was (95.33 ± 23.08) minutes (55–135 min). None of patients had heavy bleeding, mortality and conversion to open surgery during operation. There was no discomfort in fluidized diet on the first day after surgery and no postoperative complications such as delayed bleeding, fever, peritonitis, intra-abdominal abscess and umbilical incisional hernia. The umbilical incision healed well (Fig. 11) and the patients were usually discharged 4–5 days later.

### *Characteristics of GPs*

**Fig. 12:**
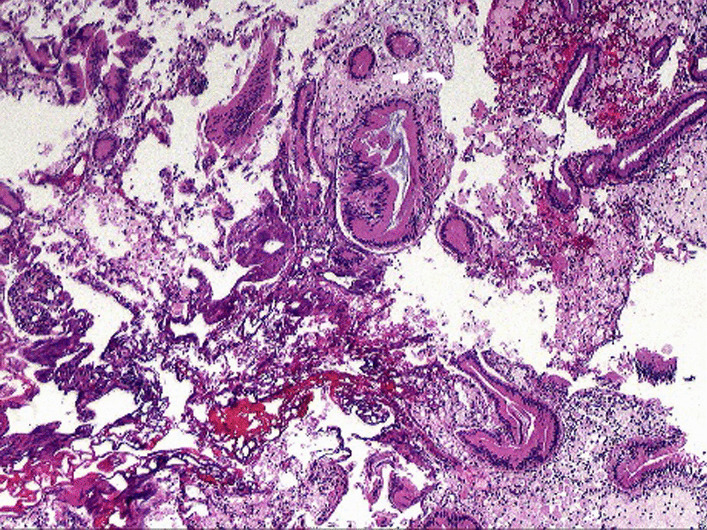
cholesterol polyps.

**Fig. 13:**
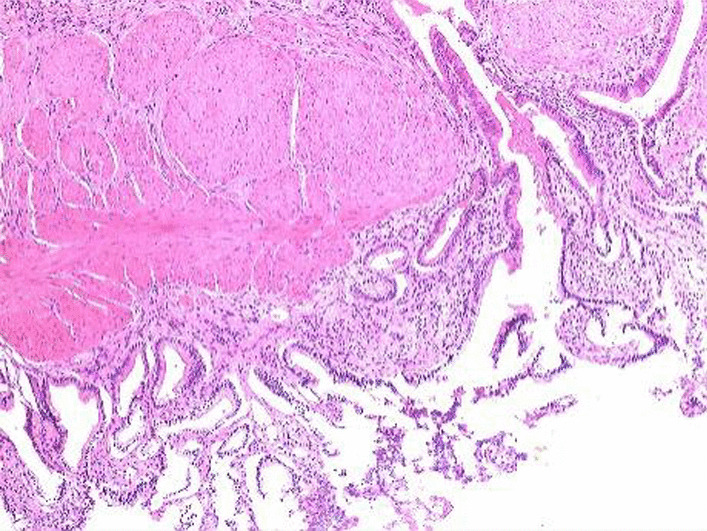
gallbladder adenomyosis

The range of GPs size was 2 mm to 15 mm, 5 cases were single polyps and 7 cases were multiple polyps. Postoperative polyp pathology confirmed that 10 cases were cholesterol polyps (Fig. 12), one of them with gallbladder adenomyosis; 2 cases were chronic mucositis (Fig. 13); no case was tubular adenomas.

### Follow-up

**Fig. 14:**
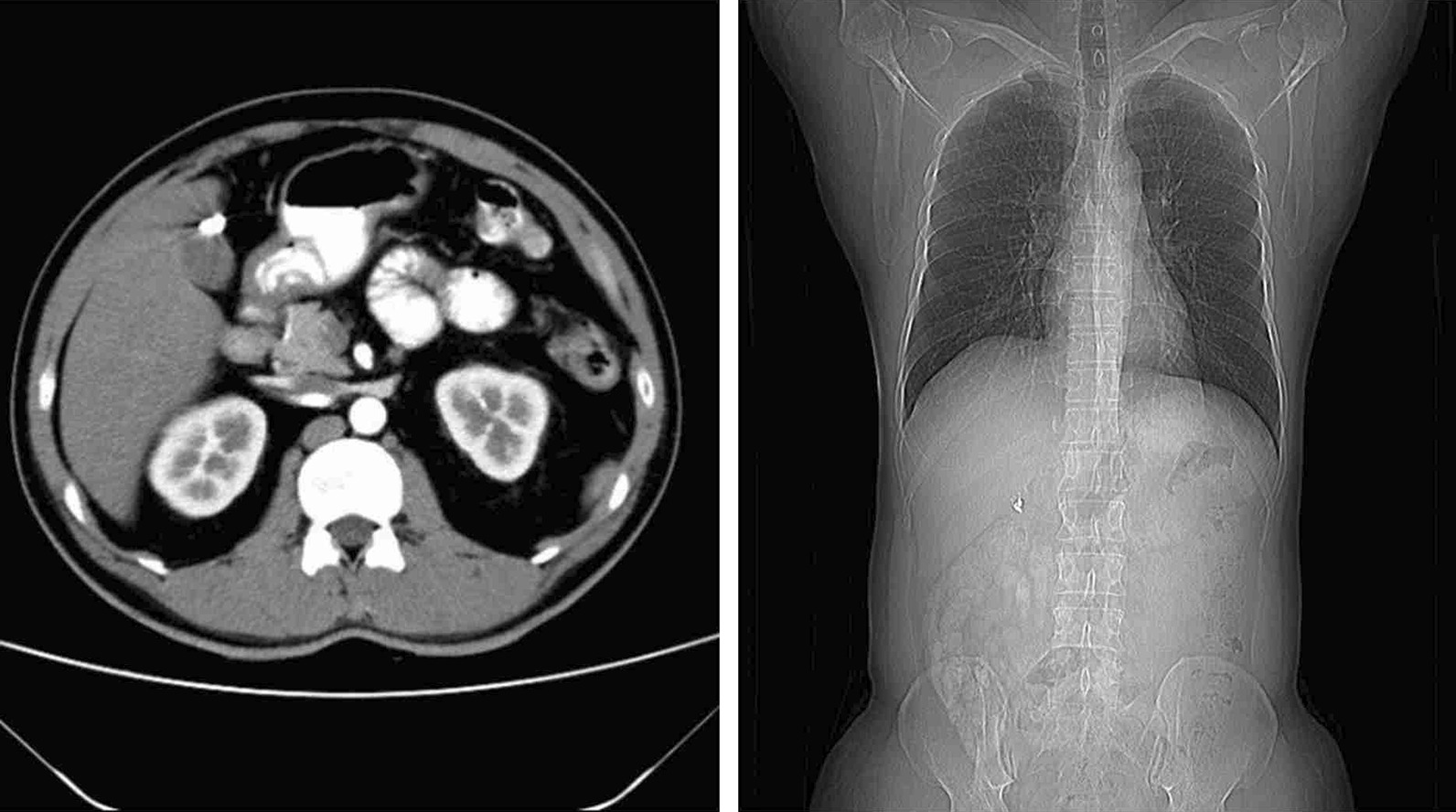
CT indicated the retention of titanium clip hemostatic clamps 12 months after surgery

All patients were followed up at 1, 3, 6, and 12 months postoperation. Follow-up showed no postoperative discomfort and no visible incision on the umbilical region in all patients, all patients were satisfied with the cosmetic results of the incision and did not experience incision pain. The GPs did not recur through abdominal ultrasonography. Hemostatic clamps did not come off through abdominal CT examination (Fig. 14).

### Gallbladder-emptying function

**Fig. 15:**
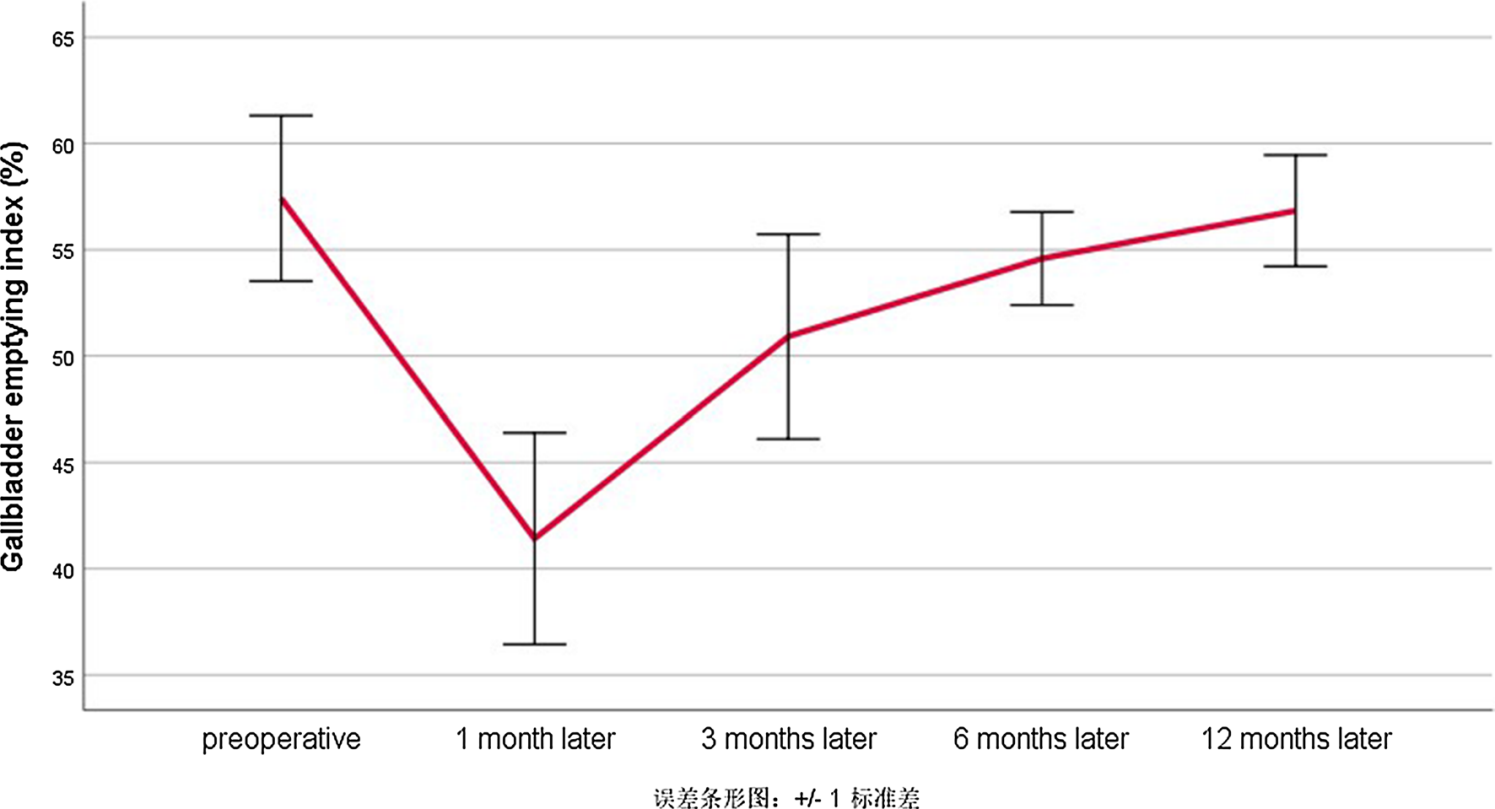
Gallbladder-emptying function before and after operation linear graph

All patients had good preoperative gallbladder emptying function (gallbladder emptying index 57.37 ± 3.93%). There was no significant difference in the fasting gallbladder volume at pre-operation and 1, 3, 6, 12 months postoperation (P > 0.05). The postprandial gallbladder volume increased significantly 1 month postoperation, and the difference was statistically significant compared with that pre-operation (P = 0.00); the gallbladder emptying index decreased 1 month postoperation, and the difference was statistically significant compared with that pre-operation (P = 0.00); the gallbladder emptying index increased 3 months postoperation and thereafter. The gallbladder emptying index reached 56.83 ± 2.83% 12 months postoperation, and there was no significant difference compared with that pre-operation (P = 0.53) (Table 1) (Fig. 15).

**Table 1:** Comparison of gallbladder-emptying function before and after operation

Time	Fasting gallbladder volume (cm^3^)	Postprandial gallbladder volume (cm^3^)	Gallbladder emptying index (%)
Pre-operation	57.04 ± 6.70	24.21 ± 2.71	57.37 ± 3.93
1 month postoperation	53.19 ± 5.65	31.09 ± 3.15	41.34 ± 4.91
3 months postoperation	54.64 ± 6.41	26.74 ± 2.80	50.80 ± 4.78
6 months postoperation	55.23 ± 6.63	24.98 ± 2.67	54.66 ± 2.28
12 months postoperation	55.90 ± 6.12	24.04 ± 2.12	56.83 ± 2.83

## Discussion

Some studies have found that gallbladder adenoma can develop into gallbladder adenocarcinoma, which is a recognized precancerous lesion [14, 15]. Gallbladder cancer is associated with a poor prognosis that the 5-year survival rate is less than 5%, the prognosis of early gallbladder cancer is significantly better than that of late gallbladder cancer [16]. So early diagnosis and early treatment is particularly important. The European guidelines recommend cholecystectomy if the diameter of GPs is greater than or equal to 10 mm [17]. However, the histology of GPs are mainly cholesterol polyps, and a few are inflammatory polyps, adenomyomas, adenomas, etc. [4]. In our study, cholesterol polyps confirmed by postoperative pathology accounted for 83.3% (10/12) of the polyp detection rates. With the reported malignant change rate of GPs at just 0.57% [18], the main surgical treatment for the diameter of GPs ≥ 10 mm consists of wide surgical and is contentious. Gallbladder is an important digestive organ of human body, which has the functions of concentrating bile, regulating bile duct pressure and regulating immunity. Blind cholecystectomy may lead to postoperative complications such as dyspepsia, alkaline reflux gastritis, colon cancer and affects patients' quality of life [19–21]. Therefore, it is necessary to find a more suitable way for the treatment of GPs.

In our study, we performed new-style E-NOTES gallbladder-preserving surgery for polyps which has its own unique advantages. Firstly, it maximizes the retention of the functional gallbladder and reduces the risk of potential cholecystectomy and postoperative biliary dysfunction. Secondly, the lesion can be completely removed to reduce gallbladder cancer risk, and the pathological diagnosis of polyps can be obtained to develop further treatment or follow-up strategies. Thirdly, compared with traditional laparoscopic-assisted gallbladder-preserving polypectomy [13, 22, 23], the main advantage of E-NOTES is that the umbilical cord is the weakest part of the abdominal wall, and transumbilical puncture can not only reduce the injury of the abdominal wall, but also relieve the pain of the surgical wound. Furthermore, the 2–4 incisions in the abdomen of the traditional laparoscopic surgery can be transformed into a small incision of 10 mm in the umbilical cord. With few scars on the body surface after the operation, E-NOTES achieve a more minimally invasive and more cosmetic effect.

Whether or not the change of gallbladder function and the recurrence of gallbladder polyps after surgery are worth exploring. We observed that the gallbladder emptying function decreased one month after surgery, and gradually improved 3, 6 and 12 months after surgery. Considering intraoperative incision of the gallbladder wall will cause postoperative gallbladder edema and scar formation one month after surgery, which may lead to postoperative adhesion and thus affect the gallbladder-emptying function. When gallbladder inflammation and edema gradually subsided 3 months after surgery, the gallbladder-emptying function gradually returned to normal. Moreover, no effect of hemostatic clamps on gallbladder-emptying function has been observed, and no discomfort caused by the presence of hemostatic clamps in the body has been found. There was no recurrence of GPs during one year follow-up.

As an emerging technique, there are no guidelines for E-NOTES gallbladder-preserving surgery for polyps, we have some tips from our practice's experience on how to operate it: ① Compared with laparoscopic technology, gastric endoscope lacks sufficient support and cannot be accurately oriented after entering the abdominal cavity. We used homemade super long Trocar to solve this problem. Trocar can guide and assist the endoscope to the operating position, and fix the endoscope to facilitate surgical operation. It should be noted that overshooting Trocar puncture has been widely recognized as a major cause of surgical injury in laparoscopic surgery [24]. Hence, Trocar should be punctured gently, so as to avoid intestinal damage or even severe bleeding caused by overexertion; ② Transparent cap-assisted endoscope should be used during the surgery, which can increase the visual field during the operation, have a fixed field of vision and leave operating space for the instruments [25], so that the gallbladder exploration and operation became smoothly; ③ There are abundant blood vessels in the gallbladder wall, so blood vessels need to be identified carefully during the cutting the gallbladder wall with HOOK knife to avoid accidental injury of blood vessels and massive bleeding. When it comes to bleeding, hot biopsy forceps should be performed timely to hemostasis; ④ 6 to 8 titanium clips were used to clamp the incision of the gallbladder wall, and the closure of the incision should be repeatedly checked to avoid bile leakage; ⑤ Before gastroscope withdrawal from the abdominal cavity, the gallbladder and abdominal cavity should be flushed with normal saline to check whether there is incision bleeding or bile leakage; ⑥ The operation is not easy, the operator should be familiar with the anatomy of gallbladder and methods of using endoscopic instruments, so the operator who have rich experience in endoscopic operation and solid surgical foundation could be competent for this operation.

The surgical indications need to be strictly grasped to reduce the occurrence of adverse reactions. For example, recurrent cholecystitis may be due to abdominal adhesion, which could limit the surgical field of vision and lead to increased operational risk or even failure of operation. Therefore, preoperative evaluation of the patient's condition and the specific characteristics of the gallbladder is very important. In our study, the absence of serious complications intraoperatively and postoperatively was due partly to some strict control of indications.

The shortcomings of this study lie in the small sample size and short follow-up time, which makes it difficultly to predict the recurrence rate of GPs in the next few years.

In summary, E-NOTES gallbladder-preserving surgery provides an effective new option for GPs. The operation is minimally invasive, safe and has significant short-term efficacy, but its long-term efficacy still needs to be studied in a large sample.

## Data Availability

The datasets used and/or analyzed during the current study are available from the corresponding author on reasonable request.
